# Lockdown and Children’s Well-Being: Experiences of Children in Switzerland, Canada and Estonia

**DOI:** 10.1007/s41255-021-00015-2

**Published:** 2021-03-29

**Authors:** Daniel Stoecklin, Christine Gervais, Dagmar Kutsar, Catrin Heite

**Affiliations:** 1 Centre for Children’s Rights Studies, University of Geneva, Geneva, Switzerland; 2 Nursing Department, Université du Québec en Outaouais, Québec, Canada; 3 Institute of Social Studies, University of Tartu, Tartu, Estonia; 4 Institute of Educational Science, University of Zurich, Zürich, Switzerland

**Keywords:** Children’s subjective well-being, Lockdown, children’s perspectives, Relational well-being, Pandemic

## Abstract

This paper addresses the well-being of children in Switzerland, Canada and Estonia, as they experienced the lockdown imposed by governments after the state of international public health emergency, declared by the World Health Organization on 30 January 2020. Suspension of school or starting with distance learning, cessation of extracurricular activities, closure of playgrounds, parks, shopping centres and loss of daily contacts with friends completely transformed children’s lives. The surveys conducted by the authors in individual ways, were all inspired by their membership to the Children’s Understandings of Well-Being network and involved the participation of 403 children aged 7–17 years old (229 girls and 174 boys). They present the emerging trends from the children’s narratives focusing on their experience of the lockdown in relation to family life, school life, contacts with friends, and in relation to space, time and self. During the lockdown leisure activities and hobbies, followed by life with friends and school life challenged relational well-being the most, while family life opened up new perspectives and generational solidarity. Staying at home and decreased physical activity impacted on the physical health of children, missing direct contacts with friends and teachers put social relations to test, fear of the virus decreased feeling safe and secure, and the lockdown restricted participation in society. The findings underline the relational nature of their well-being. More in-depth studies are needed to highlight the widening of inequalities and the balance between protection and participation of children.

## Introduction

The state of the international public health emergency, declared by the World Health Organization on 30 January 2020 and the lockdown imposed by governments had a massive impact on everyday social life and changed the lives of all families (Cluver et al. 2020). Parents and children had to deal with the situation with each other’s presence at home full-time, while adapting to changes in their working and schooling conditions and a decrease in their social interactions (Fegert et al. 2020). Suspension of school and/or the commencement of distance learning, cessation of extracurricular activities, the discontinuation of sports and leisure activities, closure of playgrounds, parks, shopping centres and loss of daily contacts with friends completely transformed children’s and parent’s lives without them necessarily being able to prepare for these changes. The sudden and unforeseen interruption of the usual routines brought uncertainty and something crisis-like into daily life, contributing to a vulnerable context for families and challenging of children’s well-being (Coyne et al. 2020; International Family Nursing Association, 2020; UNICEF 2020).

The authors of the present paper are members of the Children’s Understandings of Well-Being (CUWB) network. The rationale of the CUWB project, as presented in its research protocol, is to explore and analyse the meanings of well-being from children’s perspectives and determine how different dimensions of well-being are understood within and across national contexts (Fattore et al. 2019). This qualitative endeavour also contributes to the interdisciplinary development of indicators of child well-being (Ben-Arieh 2008; Ben-Arieh et al. 2014) and the related Children’s Worlds study (Rees et al. 2016). The latter has demonstrated that children give high estimates to general questions about subjective well-being (e.g. Rees and Main 2015; Rees et al. 2020). The lockdown put children in an unexpected new situation and challenged their well-being. However, very little is known about how they experienced this new situation being in the midst of the lockdown and receiving its direct impacts, with very little research undertaken that has listened to children. The aim of this paper is to reflect on children’s experiences during the lockdown in Switzerland, Canada and Estonia. The focus of the paper is set on changes in the well-being of children from their own perspectives during an extremely new and unexpected situation of the pandemic and the lockdown. The findings attract attention on the relationality of well-being: children’s accounts in times of Covid underlines the relational nature of their well-being. We see relational well-being as inspired by Gergen (2009), as a process of interactions of the child with his or her environments: family, friends, school and leisure, including people, relationships, time and space.

The paper first presents the scope and methodologies of the studies conducted by the authors in Switzerland, Canada and Estonia. The findings are presented in a comparative way, according to the main areas that were explored: (1) family, (2) school, (3) friends, (4) leisure. The country comparisons try to reveal some common features of the impacts of the pandemic on children doing agency and their relational well-being while also highlighting the contextual uniqueness of each country. The paper ends with recommendations for the inclusion of children’s voices in the responses to the COVID-19 pandemic and for future research on this topic.

## Introduction to the Studies in Switzerland, Canada and Estonia

Due to the sudden outbreak of the pandemic, the authors conducted qualitative studies promptly and in an individual way. It is only after the launching of their respective inquiries that they turned to their colleagues to the CUWB network to see who had done similar work in order to put their own results in a comparative perspective. Being inspired by the CUWB research protocol (Fattore et al. 2019) which they had followed for previous studies, they explored children’s experiences of the lockdown due to the COVID-19 pandemic. They all sought answers to the general question of how do children experience the time of lockdown in relation to family life, school life, contacts with friends, and leisure activities. They were also intrigued by the activities that children had to stop because of the lockdown and the new activities they had developed in relation to the unexpected situation. Altogether, 403 children aged 5–17 years of age (229 girls and 174 boys) participated in the studies. Such a large sample, which exceptional for a qualitative study, is linked to the reunion of individually prompted studies. It therefore heightens the chances to meet data saturation, central in grounded theory (Strauss and Corbin 1990). We will come back to this in the conclusion. It should meanwhile be noted that the serendipity of the process comes from the fact that the authors are part of the same research newtwork (CUWB) and are therefore sharing similar concerns and perspectives around children’s subjective life experiences.

The World Health Organisation declared a state of international public health emergency on 30 January and global pandemic on 11 March 2020. The context of the study is the Covid-19 pandemic and the effects of specific measures put in place by the governments of the 3 countries studied (Switzerland, Canada and Estonia,) to enforce the lockdown. The main differences among these countries are the following:

In Switzerland, the local governments (at the cantonal level) have priority over the national governments with regard to measures to be implemented as long as the situation is not declared “exceptional”. With the rapid acceleration of contamination (first wave) in the first months of 2020, the situation was declared exceptional and the federal government enforced a “semi-lockdown” from 13 March to 11 May 2020: the population in Switzerland could still move within and outside the country, but private contacts had to be reduced very much and the social life of the children was changed so considerably. Also schools as well as the non-essential shops and activities such as music schools or sports clubs were shut down and this also affected the children’s everyday life and leisure activities. Distance learning was put in place rapidly and the support to ease financial hardship was massive in this first wave. Hence, no major disruption had to be deplored in Switzerland in this period, while the concern for the lasting effects of the pandemic, notably in terms of commercial bankruptcy and job losses, was growing. For children living in Switzerland, the access to presential schooling and most leisure activities was impossible and their socio-spatial radius of movement as well as their contacts to peers were clearly limited. The combination of a “semi-lockdown”, the financial governmental support thanks to huge reserves of the National Bank, a relatively low and long-standing unemployment rate, and organisational efficiency in the moves towards distance learning (13 March) and back to presential schooling (11 May), impacted children’s lives in rather limited ways.

In Canada, the decisions and measures implemented regarding the pandemic are carried out by local governments (at the provincial level) and are different in every province. In Quebec, where the study was carried out, a province-wide health emergency was declared on March 14, two weeks after the first confirmed COVID-19 case. A lockdown was declared, everyone was told to stay home, schools and daycare centres were closed, and all non-essential shops and activities were suspended. People could still walk and play outside around their house but travel between regions was prohibited. Teleworking became the reality for numerous parents. Children stayed home with their parents, with no social contact and very restricted leisure activities. In response to the surge of COVID-19 cases, particularly in seniors’ residences, the lockdown was renewed on March 24 and April 5. Private schools rapidly moved to online education (after one or two weeks) but government-run schools remained closed. The peak in active COVID-19 cases was reached on May 8, 2020 (Institut national de santé publique du Québec (INSPQ) 2020). Lockdown measures were then gradually lifted, including the reopening of many shops on May 4 and the reopening with many restrictions of elementary schools and daycare centres on May 11, except for the Montreal area, which was considered too affected by the pandemic. Distance education was implemented for all secondary school students and for elementary school children in the Montreal area, which was maintained until the end of the school year on June 23, 2020 (INSPQ 2021).

In Estonia the lockdown was declared by the government on the 12th of March. The whole education system moved to distance learning, playgrounds/rooms, parks, shopping centres were closed and people were strongly advised to stay in their homes. Many hobby schools went to distance online as well. For the distant learning children needed computers and access to high-quality internet. There was a grass-root initiative to donate computers. As a result, over 1000 computers were collected and donated to families, including some brand new ones. Also, initiatives were taken to improve the internet connection. Depending on the type of work, many parents developed their home offices. According to Lauristin et al. (2020), 36% of parents used this opportunity. Many families moved home offices to their country houses with loose population and lower contamination risks. The Estonian government launched the first ‘revival package’ in March to relieve the harm of the pandemic to economy and people. The most harm was caused to the sectors of tourism and leisure and the unemployment reached almost 8 %.

The Ministry of Education and Research decided to re-open schools from the 15th of May. However, the teaching activities could start as individual consultations or in small groups (ten children as maximum) and this was addressed to children who had not had made advancements in learning during the lockdown. The others continued online. The contact hobby education re-opened from the 15th of May as well. The graduation exams of the basic school (the 9th form) were cancelled, the graduation exams for gymnasium (the 12th form) were made optional.

In Switzerland two studies were carried out. First, an online survey was run from April 21 to May 31 by the University of Geneva (Daniel Stoecklin) to shed light on the experiences of children in relation to the lockdown in the French-speaking cantons Fribourg, Geneva, Jura, Neuchâtel, Valais and Vaud. The study focused on the experiences of 157 respondents aged between 11 and 17 years regarding their family and school life, their life with friends, their hobbies, and finally their activities that were new and had they had to stop during this period. The survey was communicated and distributed by children’s services and children’s associations (mainly leisure and sports clubs) in the cantons but it was not possible, during the emergency, to rely on the departments of public education. In addition, the implementation of the survey was relatively cumbersome, as it was necessary to obtain parental consent through the above-mentioned entities. These factors limited the number of respondents in the study. The rationale behind collecting experience during the period and not after its end was the risk of losing memory of the lived experience in the light of the direct impacts of the lockdown.

Second, an exploratory study was carried out by the University of Zurich (Catrin Heite, Franziska Schlattmeier, Clarissa Schär and Katherine Bohren) involving six face-to-face narrative interviews. The study focused on different aspects of children’s everyday life during the lockdown, for example with which people, at which places and with which activities they spent their time. Methodologically following Gabriele Rosenthal’s (2018) argument for a “logic of discovery”, a detailed analysis of some sequences from the narrative of a nine-year-old girl is included in the present paper in order to show dilemmatic aspects of everyday life under the lockdown conditions. The interpretation of the interviews is thus characterized by priciples, which Rosenthal names with *reconstruction*, *sequentiality* and *abduction*. In the sense of interpretative openness, this means not approaching the analysis with a set of assumptions formulated in advance and consequently not subordinating the narration of the children to theoretically founded categories. Such an approach, which proceeds processually and not with a theory-guided hypothesis generation, works sequentially and with abductive conclusion procedures hypothesis-generating on the individual case. This makes it possible to work from the individual case in a theory-generating way and - according to Rosenthal - to enable the discovery of new, unanticipated insights. This research approach is based on an understanding of “biography” that goes beyond its sigularity and understands the human being as a subject capable of action.

In Canada, the study was conducted by the Université du Québec en Outaouais (Christine Gervais, Isabel Côté, Vicky Lafantaisie, Francine de Montigny, and Tamarha Pierce) in the province of Québec. The study aimed to provide a longitudinal description of the experiences of children and parents in relation to the COVID-19 pandemic. In total, the experiences of 192 children aged seven to 17 were collected out of which sixty interviews were selected for the present paper. The selection was based on the diversity of the participants’ personal situations, the experiences recounted, and the richness of the discourse. The fieldwork took place from April 30 to May 20, which corresponded to the peak of active COVID-19 cases in Québec (Institut national de santé publique du Québec (INSPQ) 2020) and the mandatory lockdown. Participating families were recruited via social networks (Facebook) and family organization newsletters, as well as through responding to newspaper articles and radio interviews. Parents consented to their child participating in an interview and the children’s assent was obtained using a special form developed as part of the previous work of the research team that helped to explain to children the ethical issues related to their participation (Côté et al. 2018). Children were interviewed using the Zoom platform. The interviews explored both, children’s perceptions of the lockdown, and of the future, using a scaffolding technique that promoted their active involvement and supported the construction of their explanatory and argumentative discourse, thereby maximizing their contribution in the construction of knowledge (Delamotte and Akinci 2012; Kirk 2007). The thematic analysis (Paillé and Mucchielli 2014) combined field notes and interview transcripts (Tessier 2012).

In Estonia, the study was carried out by the University of Tartu (Dagmar Kutsar and nine research practice students; interviews gathered by Kairi Laane, Julia Tross and Katariina Mikker are analysed in this paper) in different towns and a rural area. The study focused on the most recent changes in children’s lives during the lockdown. Children spoke about what was worse and what was better in their lives; how they saw their lives in general; and what would they have changed in their current everyday life if they had a magic wand. A total of 48 children participated in the study, their age ranged from five to 16 years. In this paper, interviews of 27 children of the same age range are analysed. Such a broad age scale was intentional with the aim to capture diverse patterns of children’s cognitions and feelings while staying at home during the lockdown. The interviews were carried out online or over the telephone, with a few interviews taking place face-to-face outdoors. The mixture of the data collection methods was necessitated due to the special circumstances resulting from the lockdown in Estonia. The interview transcripts and answers to the open questions obtained through the online survey were analysed using directed content analysis method. Children participated in the study on a voluntary basis and with parental consent.

## Results

Using this mix of quantitative and qualitative exploratory approaches, we identified trends in national contexts and highlight cross-national features of children’s experiences during the time of the lockdown. We then present the emerging trends from the children’s narratives focusing on their experience of the lockdown in relation to family life, school life, contacts with friends and time and space. For each theme, quantitative data collected in Switzerland are first described followed by the analyses of the qualitative data collected in the three participating countries enhancing and strengthening our understanding of children’s experiences.

### Major Trends in National Contexts

*In terms of major trends in national contexts*, the Swiss qualitative studies show that in the children’s narratives, there are representations of normal everyday life, negative changes caused by the lockdown - especially with regard to their friendship contacts - and positive aspects, which primarily relate to an everyday life that is considerably less pre-structured in terms of time, space and content. The idea that Corona is something special seems to be primarily an adult perspective and is not as pronounced in the children’s narratives. The representation of children as “healthy carriers, vectors of the virus” has placed in the background their specific needs during this social upheaval and does mean that their opinions on this crisis has been overlooked. The interviewed children seemed accepting the changing conditions induced by the pandemic with much more flexibility than their parents. Here we understand “flexibility” in psychological terms, along with the definition used by Coyne et al. (2020):

*“Psychological flexibility* refers to the ability to recognize and adapt to situational demands, to remain aware and open to the present moment such that one can recognize and shift behaviour strategies as required by situational demands, and to engage in actions that are congruent with one’s deeply held values (Hayes et al. 2012; Kashdan and Rottenberg 2010)” (Coyne et al. 2020, p. 2).

In Canada, children had a good understanding of the COVID19 pandemic and of the protective measures. There was little concern about being ill with COVID19, but many of the children were nonetheless afraid of transmitting the virus and were worried about the health of their grandparents. They reported many losses in relation to the lockdown, but also opportunities that they valued. They found it particularly difficult to be away from their friends, to be unable to attend school and the suspension of their extra-curricular activities (sports and arts), as well as the boredom that resulted from all these interruptions. On the other hand, the majority of the children reported spending more quality time with their families, as well as re-appropriating some time that they were able to use enjoy as they wished, taking advantage of this time to start new activities, take care of themselves and adopt a slower pace that suited them better.

In Estonia, the analysis revealed that children understood the seriousness of the pandemic crisis. However, they also demonstrated a positive attitude and understood why the lockdown was necessary. Social distance from friends was the most difficult challenge noted by the children. They also missed teachers and the school atmosphere and doing hobbies online could not compete with these previous contacts. Kindergarten children missed their friends and wished to return to kindergarten. However, children also gave reasons why the new situation was good for them. If they had a magic wand, they would end the virus (and the other diseases) for ever. Most often, they were concerned about the lives of their grandparents who the children understood belonged to the at-risk group for fatal outcomes from contracting the virus and whom they could not visit. Children felt that there was more anxiety and tensions in mutual relationships. Compared to girls, boys expressed more concerns about their changed everyday lives.

We now turn to cross-national comparisons about family and school life, contacts with friends, leisure activities, before finally discussing the impacts of the lockdown on children’s relational well-being.

### Trends Emerging from children’s Experiences

#### Experience of the Lockdown in Relation to Family Life

The family appears to be decisive in the lockdown experience of children. In Switzerland, 79% of the respondents declared that family life is more or less the same, 13% that family life is better than before and 8% that it has deteriorated. The overall positive evaluation of family life is shared by children from the three countries, mainly expressed in terms of more and better time spent with family members:

“*We spend more time together, we enjoy doing new things, we all learn to live together, we share the household chores, like cooking, cleaning*…” *(girl, 15 years old, Switzerland)*

*“I can spend more time with my family” (girl 13 years old; boy 10 years old, Estonia)*

*“It allowed us to spend quality time together. We will remember this time for the rest of our lives. When I have children, I will tell them about it.” (boy 9 years old, Canada)*

The lockdown is associated with more stability and opportunities to have more shared hobbies, develop greater closeness and enjoy more dialogue in the family framework. Learning new activities with their families (gardening, sewing, cooking, building, etc.) is especially popular among the children. Parents are seen as an important source of support during the lockdown period, and many said this period brought them closer to their parents:

*“(My mom and I) became really close during lockdown. We got really close, like we had the same feelings. We are really, like, connected. (...) And we helped each other during lockdown. It felt good to feel closer to my mother.” (girl 15 years old, Canada)*

However, for some children, disputes and conflicts with other family members were more frequent, and they experienced bad moods and frustration within their family as well as discomfort linked to family proximity (not being able to be alone, feeling being permanently under the observation of their parent(s)). Moreover, children also talked about time shared with their siblings during the lockdown, playing and exploring their home’s surroundings. Nevertheless, some children admitted that there was more anxiety and stress around and siblings were not always nice. Everyday life had changed in terms of activities and relationships: families lived as closed communities with more anxiety and stress and more struggles between siblings.

*“Sometimes, my family gets on my nerves. We are together a lot, and our house is big but not gigantic. So, we are stuck together a lot, and it’s a bit tiring. (girl 14 years old, Canada)*

*“I do not like that my sister and brother bully me […] Sometimes I bully them too” (boy 6 years old, Estonia)*

In somewhat of a paradox, despite these findings about staying together and sharing the same space for days with family members, some children also complained about being lonely and feeling bored. This stems from the many meaningful relationships that were put on hold during the lockdown period, for example, with extended family, such as grandparents.

*“What’s hard is when you’re alone. Your brother and sister are doing their homework, and your mother is studying, so you have nothing to do” (boy 12 years, Canada)*

*“Staying at home is so boring for me” (boy 8 years old, Estonia)*

*“I went to see my grandpa, at one point, and I was, like, talking to him on the phone and looking at him through the window, and it really broke my heart. (boy 15 years old, Canada)*

*“I cannot visit my grandparents and cannot play football with my grandma (girl 9 years old, Estonia);*

The results illustrate how the lockdown has changed intergenerational solidarity in two directions: from older to younger generations and from younger to older generations. By “intergenerational solidarity” we refer to “the different types of transfers and/or gifts occurring between persons of different age-groups and creating social cohesion between generations” (Bengston et.al., 1976 and Katz et al., 2005).

In some moments, children spoke about themselves reflecting how they did their agency at home. On one hand, they valued receiving more attention from parents and enjoyed their closeness, but they also tried to be self-directed and self-efficient and cope with the situation by themselves.

*“I like to ask help from my family when I have some problem however, I still try to manage myself” (girl 10 years old, Estonia)*

Additionally, doing agency also revealed transfers outside families between households mediated by children’s friendships, as is shown in the following case observed in Zurich. The child interviewed, given the pseudonym Liliane, narrated how she had developed a relationship with another child through the fact that her neighbour “gave” them - her, her younger brother, and the neighbouring child - a small children’s hut in the garden of the neighbouring property:

“*our neighbor [,,,] she has a small house for her children who are already grown up. So a very tiny house where the paint has already flaked off quite a bit //mhm// and then she gave it to us […] and then we decorated everything again and then we planted some flowers […] and then we have a garden in the back where we have the little bench and a small table and a chair […] there because you can sit down and read and so //mhm// and the house is also very nice and yeah” (girl 9 years old, Switzerland)*

Liliane, her younger brother and the neighbour child had opened a new place in this small house, which they appropriated as exclusively available to the three of them. The formerly dilapidated and ivy-covered children’s hut was repaired and redesigned by them - very neatly with a raked path, flowers and which they made very tidy inside. Due to the small size of the house and the small garden surrounding it, which is surrounded by a wooden fence in the same bourgeois style as the whole residential area, adults have no access to the hut and the children are very keen to keep it tidy. They enjoyed having created this order and beauty themselves - as they emphasize without the help of adults. They experienced themselves as capable subjects, who had thus created their own space, to which they withdraw to read, play, garden and paint. This case shows that opening of new “spaces of childhood” is a necessary movement that is needed by children who otherwise feel that the space usually left to them is shrinking as a result of lockdown measures. The lockdown accentuates the double-sided reality of intergenerational solidarity, namely the paradoxical social bonding in societies marked by high levels of individualism whereby one needs at the same time to belong to a group and to be recognized as a distinct self. The results about the contrasts in family life discussed previously in this section demonstrate children’s need for individual space - not only physical but also emotional and mental. The autonomy of children in the regulation of their emotional well-being could also be reduced by increased parental attention, which they sometimes perceived as exaggerated.

#### Experience of Lockdown in Relation to School

Despite that half of the respondents interviewed in Switzerland asserted that school life had not changed for them, moving to distance learning overnight was a major change for children in Estonia and Switzerland, who expressed diverse feelings concerning it. On the one hand, children valued the opportunity to plan their work, to determine the tempo and time of their activities, to be able to sleep more in the morning, be together with their family, have a meal when they felt like it, and use the internet to help them out with doing the home school exercises. Some subjects were attractive just because of the distance learning. The home atmosphere supported children to do their school exercises, in that the children were able to consult with their parents and the absence of their classmates meant that a usual source that disturbed their focus on learning in the classroom was absent. They felt they worked faster when alone in part because they did not have to wait for others to understand the instructions provided by teachers and/or the material. Some children even admitted that this was the best situation for them and had the impression that their school life had improved.

*“I can develop my time schedule” (boy 10 years old, Estonia)*

*“I can follow my own tempo doing things” (girl, 9 years old, Estonia)*

*“I like everything. I like this routine. I like the longer sleep and that there is no need to change clothes” (girl 16 years old, Estonia)*

However, several reasons were also provided as to why distance learning made children concerned and many of them experienced a deteriorated form of schooling. For children in Switzerland, the main reasons for dislike of distance learning concerned the lack of progress and the limited number of subjects covered during lockdown, while children in Estonia complained about having an increased workload from school and disliked the change in the pattern and form of communicating with their teacher. When asked about the bad things in their lives during the lockdown, the key-term children provided in their answers appeared to be ‘distance’: learning from distance and keeping friends (and dearest relatives such as grandparents) at distance, thus, children’s relational well-being was challenged.

“*I would prefer to go to school because the teacher explains in class more, now I must work more myself to understand the material” (girl 9 years old, Estonia)*

*“Sometimes when I need help from my teacher, she is not available” (girl 13 years old, Estonia);*

*“I would like to have more video talks with classmates to discuss different topics” (girl 10 years old, Estonia)*

Learning is a co-operative effort between children and teachers. Children, being used to meet their teachers on a daily basis, were no longer experiencing this routine experience, even though this was a temporary change. Moreover, distance learning also means the absence of face-to-face contacts with peers and the lack of joint activities that are important for forming the school atmosphere. They admitted that they missed school.

*“School atmosphere helped me focus on learning. At home instead there are many other things and activities that I would prefer to focus on” (girl 10 years old, Estonia).*

In Canada, schools were closed for more than 8 weeks with no distance learning except for children in private schools. Indeed, public schools were not organized for online courses and in order to avoid creating inequalities between children with computer hardware and internet access and those without access to it, governments decided to suspend all school activities during the lockdown period, corresponding to the time of data collection. In this context, children mentioned they missed their teachers and looked forward to beginning distance learning lessons to see them again, while older students, who were not going back to school “in person” before the end of the school year, tended to express disappointment about having missed the chance to say goodbye to them properly. Moreover, children also expressed great disappointment in the loss of end-of-year school activities, such as shows, graduation, or trips— important rituals that usually mark the end of the school year and the passage to another stage.

*“I miss my Phys. Ed. teacher; it’s really fun being able to do Phys. Ed. at school with him.” (girl 10 years old, Canada)*

*“For me, it’s a relief that it’s ending this way, but, at the same time, it makes me angry because it seems like I can’t say goodbye to my high school days in a legitimate way” (boy 17 years old, Canada).*

The doing of agency was clearly exhibited amongst the participants from Estonia when children stressed freedom to make time schedules themselves, do their activities at their own pace and in the order they wished. Children valued new knowledge and skills that they had developed due to the new situation and the requirements made of them through online learning. Additionally, learning online together with other children was valued because joint video-talks contributed to their relational well-being.

*“Distance learning has helped me to cope with the computer’s world, this is new and cool experience” (girl 9 years old, Estonia)*

*“I like doing my exercises in the computer. I like video-talks” (boy 8 years old, Estonia)*

*“Video-talks are good because at least then I can communicate with friends” (boy 11 years old, Estonia).*

Children acknowledged the value of conventional face-to-face contact learning at school and seeing friends face-to-face as an engine of their relational well-being. Still, the pandemic brought about dramatic change in the customary learning process for all parties. The question remains whether communication from distance and the home environment would have a similar potential for reaching the joint aims of learning and personal growth. Moreover, can distance learning compensate for the missing face-to-face communication and safeguard the relational well-being of children, teachers and parents.

#### Experience of the Lockdown in Relation to Contact with Friends

Half of the respondents in Switzerland said that their social life with friends stayed more or less the same. Only 8 % said it was better than before, and 42% complained about the situation getting worse. Separation from friends or loss of contact with them is also mentioned as a significant difficulty in lockdown in the qualitative research with the children in the three countries. Family isolation and distancing were felt as the cause for the decline in the quality of friendships:

*“We only see each other through video calls, so it’s more difficult to talk about personal matters and we are suddenly less close than before.” (girl 13 years old, Switzerland)*

Social distance was perceived as negatively affecting friendship because it led to less dialogue and less things to share with friends, leaving children with hardly any possibility for seeing each other in person. Some explained that it was not the same as before the lockdown with their friends— that they do not know what to say to them, as they no longer share their daily lives, do not really know how to keep in touch, and miss playing in a group. This kind of social distance seems especially difficult for adolescents who found it very difficult to no longer be able to see their romantic partner. In fact, finding other means for communication could not substitute for real contact.

*“I don’t think I am experiencing joy, like, not at all. I can’t see my friends, and time passes slowly.” (boy 11 years old, Canada)*

*“Communication with friends over social media is not enough for me, I miss direct contacts with friends” (girl 16 years old, Estonia)*

*“I would like to spend time with my friends, to speak with them and play” (girl 11 years old, Estonia)*

Preschool children also said they missed their friends and kindergarten teachers, and were waiting for the time to go back to kindergarten:

“*I cannot see my friends, we would play together and I would show them my new things. I am so sad that I cannot meet my friends” (girl 5 years, Estonia)*

Friends are important factors in determining relational well-being of children. This becomes very clear in the narratives where children speak about missing their (best) friends. This missing is described as a strong feeling, which is also reflected in their concrete everyday life. At the same time, this is also where the possibility of something new becomes apparent: the fact that the fixed structure of spending time with the (best) friends is eliminated by lockdown also leads to the creation of new individual friendships, evident in “corona relationships” being formed between two to three families in the neighbouring area. For example, Liliane, the 8-year-old girl, reports the following:

*"I also like the fact that I had contact with other children with whom I didn't play with before because I always played with my best friend. I haven't played with her for a long time //mhm // and then I thought it was cool to do something with the other girl". (girl 9 years old, Switzerland)*

Because friendships - and especially “best friendships” - have an excluding character and this character is now reduced by the pandemic – regulation, this has two connected consequences. On the one hand, Liliane finds it sad that she cannot meet her two best friends at the moment and that the virtual contact she has with them is also insufficient. At the same time, she evaluates it as very positive that she has developed a new friendship precisely for this reason - the social time-space void that is filled by a child who she still knows from her kindergarten days, but who had not yet been one of her friends.

#### Experience of the Lockdown in Relation to Space, Time and Self

In Switzerland, 48% of the respondents mentioned that their leisure time was negatively impacted by the semi-lockdown. For 30%, their hobbies were more or less the same and 21% mentioned an improvement. Those who found their hobbies had improved said that they had more time for leisure/hobbies (e.g., music, crafts, etc.). They also mentioned the opportunity to do different activities that are more creative, doable alone, and a greater freedom to choose and schedule the activities. Many children found that they appreciated the growing shared hobbies and activities with family members. Canadian children mentioned the same.

*“I’m learning to cook and to have fun cooking. We also have time to take care of our garden. We planted lots of seedlings; we have lots of peppers, tomatoes. We also planted flowers. We have time to discover new things.”* (girl 15 years old, Canada).

In an opposite set of experiences, the negative evolution of leisure activities is linked to restrictions, such as being able to do less sport (interruption of lessons and competitions), less outdoor recreation because they are not feasible to do inside, and having less leisure time with friends. Hobbies and leisure pose the greatest problem for respondents, who found themselves not only being deprived from the activities they enjoy but also from the social bond that these activities allow. They described feeling less physically well since they had stopped practising their sports, sleeping less well, not knowing how to train alone, and being worried about regaining their physical condition after lockdown. Children listed many spaces and hobbies to which they no longer had access and which they missed.

*“(What I would like) is to be able to go to places like skateparks, skate with my friends… we can’t go now because it’s closed, and there are roadblocks.” (boy 10 years old, Canada);*

*“…we have to keep our distance, and we can’t go to public places.” (girl 14 years old, Canada)*

Similar findings were evident in Estonia, where during the lockdown some extra-curriculum activities closed doors while some continued online: children had their music and singing, and even competitive dance e-classes that added indoor work load to children at home. In the interviews, children expressed feeling sorry especially for missing outdoor games with friends and did not speak much about their hobby at schools.

Along with missed and cancelled activities, lockdown also inspired the discovery of new activities which allowed them to adopt new routines that were more consistent with their own rhythm and to take more care of themselves (e.g., doing yoga, writing positive thoughts in a journal, etc.). In Switzerland, for the new activities (family activities, games, sports), two thirds of the children made decisions themselves about what the new activities were. The activities that had been imposed on them mainly concerned household chores - children thus showed only some spirit of initiative. For activities that children had to give up - those involving close contact: mainly sports, dancing, seeing friends, going to town, to restaurants, etc. - impediments were imposed in the majority of cases (75%). This shows that children have agency in designing the new activities they want to have, while they are to obey the restrictions imposed on them. The collective dimension of new joint activities has to be underlined: children particularly appreciated initiating new activities with their families (gardening, sewing, cooking, building, etc.). However, it seems to predominate that the children appreciate having more time for themselves and to do activities such as reading and playing. Here it seems to be above all about an appreciation of significantly more possibilities of temporal self-direction and developing their own agency. Taking time for themselves obtained a real value in children’s perspectives.

*“I found out that I like climbing trees, since we have a forest in front of our house, and my brothers and I go into the woods more.”* (boy 12 years old, Canada)

*“But now we are freer, we enjoy life more, we enjoy the present moment more— that’s it.” (girl 10 years old, Canada);*

The lack of activity allowed a slower pace and helped children to feel less stress and anxiety. This newly discovered freedom during lockdown allowed them to learn about themselves and to appreciate the small pleasures of their daily lives.

In the present studies, children thus reported ambivalent experiences in terms of hobby activities and leisure. On the one hand, there were restrictions in their local area: the apartment, the house, the garden, the street, the nearby forest. The compulsion not to be able to leave this space was experienced as restricting and limiting. However, children tried to explore their experiences and learn from negative ones, for example how to cope without meeting friends or spend time outdoors.

*“I actually realized that I wasn’t necessarily benefiting enough from what I had because I took it for granted.”* (boy 17 years old, Canada)

*“I think this is mentally challenging how you cope with doing nothing, staying all the time in the same place, not to get tired from all this (girl 16 years old, Estonia)*

*“I do not understand my new experience whether this is related to the lockdown. This is like I am learning working more independently, I learn not to meet others” (girl 11 years old, Estonia)*

Children reported completely new appropriations of space, new experiences and possibilities in this space - such as Liliane’s narration about the children’s hut in the neighbouring garden. The situation is somewhat clearer with regard to time: here, the children also admit the lack of a time structure, boredom and loss of time.

Finally, children shared ideas how to make their lives better, thinking of not only about themselves but also about other people. Moreover - being concerned about the entire World. The new life situation had caused some hesitation and changes in their worldview.

*“I could make my life better in the current situation. For example, if I could think that not spending time outdoors or not going for shopping is for everybody’s good (girl 10 years old, Estonia)*

*“There are two poles inside myself. One of them is thinking, oh, I would wish there would be peace in the world, no racism. My other pole thinks of money, clothes, cars… World peace, no global warming, and then … please ten “milkies” to me and everything will be ok” (girl 16 years old, Estonia).*

To conclude, children were poised to develop their agency through coping with new unexpected situations, acting more independently, taking responsibility and thinking of the value of other people (e.g., relatives, friends, teachers, etc.). They exercised their relational well-being in new ways and applied this in different aspects of their life.

## Discussion and Conclusions

Similar findings emerged from the studies, showing that the well-being of children is relational. Our findings about children’s experiences of measures to contain the Covid-19 pandemic magnify the relationality of well-being: as a process of interactions of the child with his or her environments (family, friends, school and leisure, including people, relationships, time and space), it was affected by lockdown measures in both positive and negative ways. For instance, the impossibility of doing certain activities was experienced painfully while new possibilities for spending time opened up. Thus, new life situations uncovered both – new vulnerabilities and opportunities for children. Children clarified how their relationships with parents were affected by the lockdown: some children experienced much support from parents and enjoyed great family times; some others reported being left alone or had to cope with distressed and overloaded parents; some children experienced more intense parental control as an impediment to their autonomy. In a similar way, some children experienced a rapprochement with their brothers and sisters through new games and shared moments, while others faced conflicts and felt the lack of their own space and privacy. Moreover, children described how their customary spaces, that allowed for a diversity of relationships prior to the lockdown, were hardly maintained during the lockdown. The reduction of contacts with friends and the shift to on-line exchanges made children long for their friends. However, missing some regular relationships or activities and having more free time led them to discover new things in an autonomous and self-determined way, thus demonstrating some flexibility of their relational well-being.

We noticed a greater flexibility with children in adapting their lives to these new conditions than has been suggested in studies on parents’ well-being during the COVID-19 pandemic. Indeed, a National Survey in the United States highlighted a deterioration of mental health in 27% of the parents surveyed during the first months of the pandemic (Patrick et al. 2020). In a similar way, Russel’s study identified that the caregiving roles and responsibilities assumed by parents during the lockdown contributed to an increase in their anxiety and depressive symptoms as well in their perceptions of their children’s stress. This, in turn, contributed to parent-child conflicts and decrease in closeness (Russell et al. 2020). The COVID-19 pandemic seems to be a particularly stressful experience for parents, as the uncertainty and unpredictability of the situation and its effects on them and their children have a strong impact on their well-being (Gonzalez and MacMillan 2020). On the other hand, children who participated in our studies report both negative and positive experiences during the lockdown.

While no differences in the lockdown experience according to the age of the participants emerged from our analyses, the wide age range of our participants (6–17 years old) possibly contributes to this greater flexibility we identified. Indeed, some recent study reported that young people used different coping strategies during the lockdown depending on their ages (Domínguez-Álvarez et al. 2020). It is possible that school-aged children, as reported by Domínguez-Álvarez and colleagues, used more problem-solving and social support coping strategies, which contributed to their discoveries and the shared activities with their brothers, sisters and parents they narrated as to their relational well-being during the lockdown. On the other hand, coping strategies of older children, engaged in more self-oriented strategies as positive emotion regulation and wishful thinking as the authors suggested, could contribute to a lockdown experience characterized by the lack of privacy and activities as well of loneliness, that was reported by some of our participants. Meanwhile, the extent of the resilience of children in the pandemic is still to be researched, as well as the most effective coping strategies to deal with this unprecedented situation.

The lockdown seems to accentuate inequalities – the increased involvement of parents in distance learning education increases the effect of the hidden curriculum, that is to say educational inequalities linked to the differential possession of economic, social and cultural forms of capital (Bourdieu and Passeron 1970). With the collective learning mode disappearing, multilateral exchanges in the classroom, a fundamental dynamic of learning by comparison, grasping nuances and concerted adhesions, are sorely lacking and social constructivist ways of learning are limited. Families differ in the material (extra) resources they have available to them, the need of which emerged as an additional source of inequality between children. For example, in an advantaged family in which the only child has his or her own computer and attends classes with the regular assistance of one or both parents, we can see that the ability to deal with lockdown draws upon a range of economic, cultural temporal and interpersonal resources available within the household. In contrast, the situation looks different when parents also work from their home offices and the need for computers increases. Moreover, there are families with several children where children have to compete for access to the family’s only computer. With teaching that takes the form of “assisted homework”, the assistance available to the pupil at home becomes a very important factor for success and a source of increasing educational inequality because of the children’s dependence on parental skills to help a child. In Estonia children complained about teachers not explaining enough or the requirements were not clear enough for children or children did not like the ways in which home exercises had to be submitted. The latter situation also creates ethical problems relating to maintaining privacy for the child. This is illustrated in a situation related by one of our participants, who confessed that she disliked that the mother submitted videos of home exercises to teachers with her face displayed. However, the child did not have another choice to meet the teacher’s requirements.

The Estonian Forum of Education, in cooperation with the Ministry of Education and Research, carried out an online survey among children, teachers and parents just before the end of the lockdown (May 12–182,020). In this survey, 686 students, 515 parents and 338 teachers responded to this pilot (not representative) quantitative study. The study revealed that about one third of students (37%) preferred distance learning and 28% preferred contact learning while 35% did not prefer one over the other (Lauristin et al. 2020). The study showed that students who did not prefer distance learning had lower self-direction capacity (including self-regulation, self-motivation and self-reflexion), and they missed the motivation provided by the school environment and communication to a larger extent than other students surveyed. Those preferring distance learning valued its potentials for exercising agency: they could choose their own tempo and schedule of activities with lower distress, thus being self-directed.

From our research, we found that during the lockdown, leisure activities and hobbies, followed by life with friends and school life challenged relational well-being the most, while family life opened up new perspectives and generational solidarity. Beyond the nuances observed within these areas, what does this tell us about how children feel? Our findings indicate a reduction in freedom due to lack of contact (with friends, through leisure): in other words, autonomy is not inversely proportional to constraints because friendship and leisure are also strongly standardized. On the contrary, autonomy and relational well-being develop through cooperation, and this requires contact and proximity.

If we return to the formulation of the notion of well-being provided by Andrews et al. (2002), our study demonstrated that staying at home and decreased physical activity can impact on the physical health of children; that missing direct contact with friends and teachers can put social relations to test; that fear of the virus decreased children feeling safe and secure; and that the lockdown in general restricted participation in social life. Together, these factors created new vulnerabilities in children’s lives: they modified children’s everyday routines and endangered their relational well-being, putting its flexibility to test. Meanwhile, our results also show that the interviewed children exerted agency in using free time in more autonomous ways. Some even report feeling less stress, being in a better mood or having more time to think about their well-being during the lockdown. In addition, the emotional discomfort mentioned by some (depression, loneliness, isolation) seems more be linked to the restrictions put in place during lockdown than to the health situation itself. The social arrangements mediating and mitigating exposure to a common danger may sometimes be more problematic than the actual danger itself (Covid-19 seems not perceived with much anxiety by children, maybe because it is less severe with children). This paradox can be explained by shrinking autonomy, which actually also reveals its relational dimension: autonomy, in fact, is a relational issue. The lockdown hence reveals some hidden pattern of intergenerational solidarity, namely that it truly develops not when it is just a set of prescriptive roles and functions, but when new “spaces of childhood” are opened along the recognition of innovative competences and new forms of belonging. Restrictions to see friends, coupled with more surveillance from parents, are on the opposite impairing children’s inventiveness and collective agency.

### Critical Look into the Present Study and Recommendations for Future Research

The authors observed a great deal of diversity (items negatively and positively appreciated) in the experiences of children during the (semi-) lockdown measures. The lockdown exacerbated the cumulative disadvantages and inequality of children and thus endangered their relational well-being. But it also fostered children’s autonomy and agency. The limits of the present study are linked to the time constraints that obliged the researchers to elaborate on their respective protocols and collect data in a very quick way. More research is needed, and protocols should be adapted, especially to reach marginalised children, who are under-represented in this study because of the limitations we faced in accessing them through schools and associations that were due to the peculiar sanitary measures and hence constraints on the study. Some more in-depth studies are needed to highlight the widening of inequalities and its effect on the flexibility of children’s well-being and their resilience. More generally, the apparently higher flexibility of children in terms of adaptation to the situation, compared to adult discourses (Coyne et al. 2020), needs to be further scrutinized, especially as “psychological flexibility” can be considered an important constituent of well-being.

Studies are also needed to further understand how autonomy develops through cooperation, and how restrictive public health measures affect this process, sustaining the relational turn in childhood studies that already departed from the individualistic ideology and the myth of the individual child (Oswell 2012). Accordingly, the balance between protection and participation rights of children could be specified. Meanwhile, the features identified in this paper can be refined through data saturation, as developed in grounded theory (Strauss and Corbin 1990). This implies that all the facets of a given phenomenon are reflected when no further interview highlights any new aspect of that phenomenon. Once qualitative saturation is reached, it is possible to develop a quantitative survey to capture the statistical prevalence of the aspects identified in the qualitative work. The present paper is therefore just a starting point in this endeavour. The analysis conducted here is a first “mapping” of common features.

## Data Availability

Each author has stored their respective data in the institutions they are affiliated to. Code Availability Not applicable.
